# Astragaloside IV Suppresses High Glucose-Induced NLRP3 Inflammasome Activation by Inhibiting TLR4/NF-*κ*B and CaSR

**DOI:** 10.1155/2019/1082497

**Published:** 2019-02-19

**Authors:** Bin Leng, Yingjie Zhang, Xinran Liu, Zhen Zhang, Yang Liu, Hongxin Wang, Meili Lu

**Affiliations:** ^1^Key Laboratory of Cardiovascular and Cerebrovascular Drug Research of Liaoning Province, Jinzhou Medical University, Jinzhou 121001, China; ^2^First Affiliated Hospital of Jinzhou Medical University, Jinzhou 121001, China

## Abstract

Long-term exposure to high glucose induces vascular endothelial inflammation that can result in cardiovascular disease. Astragaloside IV (As-IV) is widely used for anti-inflammatory treatment of cardiovascular diseases. However, its mechanism of action is still not fully understood. In this study, we investigated the effect of As-IV on high glucose-induced endothelial inflammation and explored its possible mechanisms. In vivo, As-IV (40 and 80 mg/kg/d) was orally administered to rats for 8 weeks after a single intraperitoneal injection of streptozotocin (STZ, 65 mg/kg). In vitro, human umbilical vein endothelial cells (HUVECs) were treated with high glucose (33 mM glucose) in the presence or absence of As-IV, NPS2143 (CaSR inhibitor), BAY 11-7082 (NF-*κ*B p65 inhibitor), and INF39 (NLRP3 inhibitor), and overexpression of CaSR was induced by infection of CaSR-overexpressing lentiviral vectors to further discuss the anti-inflammatory property of As-IV. The results showed that high glucose increased the expression of interleukin-18 (IL-18), interleukin-1*β* (IL-1*β*), NLRP3, caspase-1, and ASC, as well as the protein level of TLR4, nucleus p65, and CaSR. As-IV can reverse these changes in vivo and in vitro. Meanwhile, NPS2143, BAY 11-7082, and INF39 could significantly abolish the high glucose-enhanced NLRP3, ASC, caspase-1, IL-18, and IL-1*β* expression in vitro. In addition, both NPS2143 and BAY 11-7082 attenuated high glucose-induced upregulation of NLRP3, ASC, caspase-1, IL-18, and IL-1*β* expression. In conclusion, this study suggested that As-IV could inhibit high glucose-induced NLRP3 inflammasome activation and subsequent secretion of proinflammatory cytokines via inhibiting TLR4/NF-*κ*B signaling pathway and CaSR, which provides new insights into the anti-inflammatory activity of As-IV.

## 1. Introduction

Diabetes mellitus is the third leading cause of death in China, and only 25.8% of the diabetes patients received treatment for diabetes [1]. Chronic hyperglycemia is a major feature of diabetes, and it is the main initiator of diabetic vascular complications [2]. Hyperglycemia alters endothelial cell function and metabolism, which in turn causes vascular damage. The damaged vascular endothelium contributes to the development of diabetic complications, especially vascular complications [3]. Multiple mechanisms are involved in the development of diabetic vascular complications, including inflammation [4].

Martinon et al. first proposed the concept of “inflammasome” in 2002 [5]. Among the inflammasomes, the NLRP3 inflammasome is currently the most studied and is a polyprotein proinflammatory complex consisting of NLRP3, apoptosis-associated speck-like protein (ASC), and pro-caspase-1 in cytoplasm [6]. Assembly of an inflammasome ultimately results in the autocatalysis and activation of pro-caspase-1 into active caspase-1. Once activated, caspase-1 cleaves pro-IL-1*β* and pro-IL-18 into mature IL-1*β* and IL-18 [7, 8]. NLRP3 inflammasome activation is involved in the pathogenesis of cardiovascular diseases, including atherosclerosis [9, 10], diabetic cardiomyopathy [11], viral myocarditis [12], ischemic stroke [13], and vascular endothelial dysfunction [14]. Activation of the NLRP3 inflammasome requires activation of TLR4/NF-*κ*B signaling pathways; subsequently upregulates inflammasome components, including inactive NLRP3, pro-IL-1*β*, and pro-IL-18; and then assembles ASC, NLRP3, and pro-caspase-1 into a polyprotein complex [6]. Calcium sensing receptor (CaSR) belongs to the G protein-coupled receptor family and plays a key role in Ca2+ homeostasis and in the pathophysiology of cardiovascular disease [15]. In addition, CaSR also plays an important role in apoptosis, proliferation, hormone secretion, differentiation, and migration [16–18]. As the promoter and responder of the inflammation [19], activation of the CaSR is associated with the occurrence and development of vascular calcification, uncontrolled blood pressure, atherosclerosis, and hypertension [20, 21]. Meanwhile, CaSR also participates in the activation of the NLRP3 inflammasome [22].

*Astragalus membranaceus* is a traditional Chinese herbal medicine; its effective ingredient Astragaloside IV (As-IV) is widely used in the treatment of cardiovascular diseases, including antimyocardial hypertrophy [23], antimyocardial fibrosis [24], antihypertension [25], and antiatherosclerosis [26]. Although As-IV has a strong anti-inflammatory effect [27, 28], its molecular mechanism remains to be elucidated. Therefore, in the present study, we analyzed the expression and distribution of TLR4, nucleus NF-*κ*B, CaSR, and NLRP3 inflammasome in vivo and in vitro in order to determine whether As-IV inhibits the activation of the NLRP3 inflammasome through the TLR4/NF-*κ*B pathway and CaSR and exerts anti-inflammatory effects.

## 2. Materials and Methods

### 2.1. Chemicals and Reagents

Astragaloside IV (HPLC ≥ 98.0%, Cat No. JZ16042403) was purchased from Nanjing Jingzhu Bio-Technology Co. Ltd. (Nanjing, China). Dulbecco's modified Eagle's medium (DMEM, Cat No. SH30021.01) was purchased from HyClone (Logan, Utah, USA). CaSR-overexpressing lentiviruses were purchased from Shanghai GeneChem Co. Ltd. (Shanghai, China). TLR4 small interference RNA (siRNA) transfection reagents (Cat No. sc-40260-SH) were purchased from Santa Cruz Biotechnology (Shanghai, China). Fetal bovine serum (FBS, Cat No. 11011-8611) was purchased from Tianhang Biotechnology (Zhejiang, China). Streptozotocin (STZ, Cat No. S0130) and ASC (Cat No. PRS2287) were purchased from Sigma-Aldrich (Shanghai, China). NPS2143 (CaSR inhibitor, Cat No. S2633) and INF39 (NLRP3 inhibitor, Cat No. S8559) were purchased from Selleck (Houston, USA). BAY 11-7082 (NF-*κ*B Inhibitor, Cat No. M2040) was purchased from Abmole Bioscience (Houston, USA). TLR4 (Cat No. 19811), caspase-1 (Cat No. 22915), IL-18 (Cat No. 10663), I*κ*B*α* (Cat No. 10268), *β*-actin (Cat No. 66009), and Histone H3 (Cat No. 17168) were purchased from Proteintech (Wuhan, China). NF-*κ*B (Cat Nos. ab38054 and ab21014) and CaSR (Cat No. ab29236) were purchased from AbSci (Nanjing, China). NLRP3 (Cat No. ab214185) and IL-1*β* (Cat No. ab9722) were purchased from Abcam (Cambridge, UK). Human IL-18 and IL-1*β* ELISA kits (Cat Nos. m1027422 and m1028592, respectively) and rat IL-18 and IL-1*β* ELISA kits (Cat Nos. m1002816 and m1037361, respectively) were purchased from Mlbio (Shanghai, China). Nuclear and cytoplasmic protein extraction kit (Cat No. P0027) was purchased from Beyotime Biotechnology (Nantong, China).

### 2.2. Animals and Treatments

Male Sprague Dawley rats (200-250 g) used in this study were purchased from the Experimental Animal Center of Jinzhou Medical University (Jinzhou, China). Experiments on animals followed the Guide for the Care and Use of Laboratory Animals published by the US National Institutes of Health (NIH publication no. 85-23, revised 1996), and all animal treatment protocols for this study were approved by the Animal Experimentation Ethics Committee of Jinzhou Medical University. A single intraperitoneal injection of STZ (65 mg/kg) was used to establish the diabetic model. 7 days after STZ injection, the blood glucose level above 16.7 mmol/L was considered as diabetic. Then, diabetic rats were randomly divided into 3 groups (*n* = 8): the diabetic group, As-IV 40 mg/kg group, and As-IV 80 mg/kg group. The normal and diabetic groups were given 0.5% CMC-Na, and As-IV groups were given As-IV 40 and 80 mg/kg, respectively, by intragastric administration. After 8 weeks of As-IV treatment, the rats were anesthetized with 20% urethane and then sacrificed. After killing the rats, blood samples were collected via cardiac puncture, and the thoracic aorta was removed for western blot and immunofluorescence staining.

### 2.3. Cell Culture

Human umbilical vein endothelial cells (HUVECs) were obtained from KeyGen Biotech (Nanjing, China). HUVECs were cultured in DMEM containing 10% (*v*/*v*) FBS and 100 U/mL penicillin/streptomycin at 37°C in an environment with 5% CO_2_. HUVECs were treated with NPS2143 (N, 100 nM), BAY 11-7082 (B, 5 *μ*M), and INF39 (I, 10 *μ*M) for 30 min following the addition of glucose (HG, 33 mM) incubation present or absent of As-IV (50 *μ*M and 100 *μ*M) for 48 h.

### 2.4. Lentiviral Overexpression of CaSR in HUVECs

HUVECs were seeded into 24-well plates (1 × 104 cells/well) overnight, and then cells were infected with 500 *μ*L of enhanced infection solution containing 5 *μ*g/mL of polybrene and 5 *μ*L of lentiviral vector (titer = 2 × 108 TU/mL) to enhance the CaSR expression in HUVECs (LV-CaSR) according to the manufacturer's instructions. The untransduced cells (control, 5.5 mM glucose) or the empty vector lentivirus (LV-Con) was prepared as controls in the experiments. At 12 h after infection, fresh medium was replaced and maintained for a further 72 h. CaSR-transduced cells were then incubated with 10 *μ*g/mL of puromycin in culture medium for 4 days to select stably transfected cells. After the selection, the HUVECs infected with or without LV-CaSR were expanded. Overexpression of CaSR was assessed using western blot.

### 2.5. ELISA

The levels of IL-18 and IL-1*β* protein in plasma and HUVEC supernatants were determined using commercially available enzyme-linked immunosorbent assay kits according to the manufacturer's instructions.

### 2.6. Immunofluorescence Staining

5 *μ*M of the paraffin-embedded tissue was deparaffinized in xylene and rehydrated in graded ethanol (100, 95, 90, and 80%). Antigen was retrieved by 10 mM sodium citrate buffer at 121°C for 3 min. After natural cooling, slides were permeabilized with 0.1% Triton X-100 in PBS for 15 min and then incubated with 5% bovine serum albumin in PBS for 30 min. Next, the slides were incubated with the primary antibody anti-NF-*κ*B p65 (1 : 100) at 4°C overnight, followed by incubation with the fluorescein isothiocyanate- (FITC-) conjugated goat anti-rabbit secondary antibody at room temperature for 1 h in the dark, and finally, nuclei were stained with DAPI for 2 min. Fluorescence images were collected using a fluorescence microscopy (Leica Microsystems).

Cells were seeded in 96-well plates and incubated for 48 h. Cells were then fixed with 4% paraformaldehyde in phosphate-buffered saline (PBS) for 15 min at room temperature and washed three times with PBS to stop fixation. The latter protocol is the same as animal immunofluorescence.

### 2.7. Extraction of Cytoplasmic and Nuclear Proteins

Cytoplasmic and nuclear protein fractions were extracted by a protein extraction kit according to the manufacturer's instructions. Finally, the supernatant was collected and was used for western blot analysis.

### 2.8. Western Blot

The collected thoracic aorta and HUVECs were homogenized in ice-cold RIPA lysis buffer, and the lysates were centrifuged at 12,000 g at 4°C for 20 min, then the concentration of protein in the upper layer of the solution was determined by using the BCA Protein Assay Kit (Beyotime Biotechnology, China). 40 *μ*g of protein was separated by 8-12% SDS-PAGE (2 h, 85 V) followed by transfer onto the PVDF membrane (GE Healthcare Life Sciences, USA) using semidry methods (22 V, 15 min). Membranes were blocked in 1% (*w*/*v*) bovine serum albumin (BSA) in 0.1% Tween 20 in TBST for 1.5 h at room temperature and incubated with primary antibodies against IL-18, IL-1*β*, NLRP3, ASC, caspase-1, CaSR, TLR4, NF-*κ*B, I*κ*B*α*, Histone H3, and *β*-actin overnight at 4°C. The next day, the membranes were incubated with secondary antibodies in TBST buffer at room temperature for 2 h. The protein bands were visualized using a Super Western ECL kit (Future Biotech, China), and the intensity of protein was quantified using NIH ImageJ software. The results were normalized to *β*-actin or Histone H3.

### 2.9. Small Interference RNA

In order to assess the role of TLR4, we used siRNA to silence the expression of TLR4. Scramble siRNA was used as control (si-Con). HUVECs were transfected with siRNA for TLR4 or scramble siRNA using Lipofectamine 2000 according to the manufacturer's protocols. After 24 h incubation with TLR4 siRNA, the cells were cultured with high glucose for an additional 48 h. The cells were divided into 3 groups: the scramble siRNA control group (5.5 mM glucose, si-Con), si − Con + high glucose (33 mM glucose, si-HG), and si − HG + si − TLR4 (si-TLR4).

### 2.10. Statistical Analysis

The data are presented as the mean ± SD of at least three repeating times and analyzed using SPSS 23.0 (IBM). Statistical analysis was performed using one-way ANOVA. Significance was defined as *P* < 0.05 or *P* < 0.01.

## 3. Results

### 3.1. As-IV Inhibited NLRP3 Inflammasome Activation and Subsequent Proinflammatory Cytokine Secretion in the Aorta of Diabetic Rats

**Figure 1:**
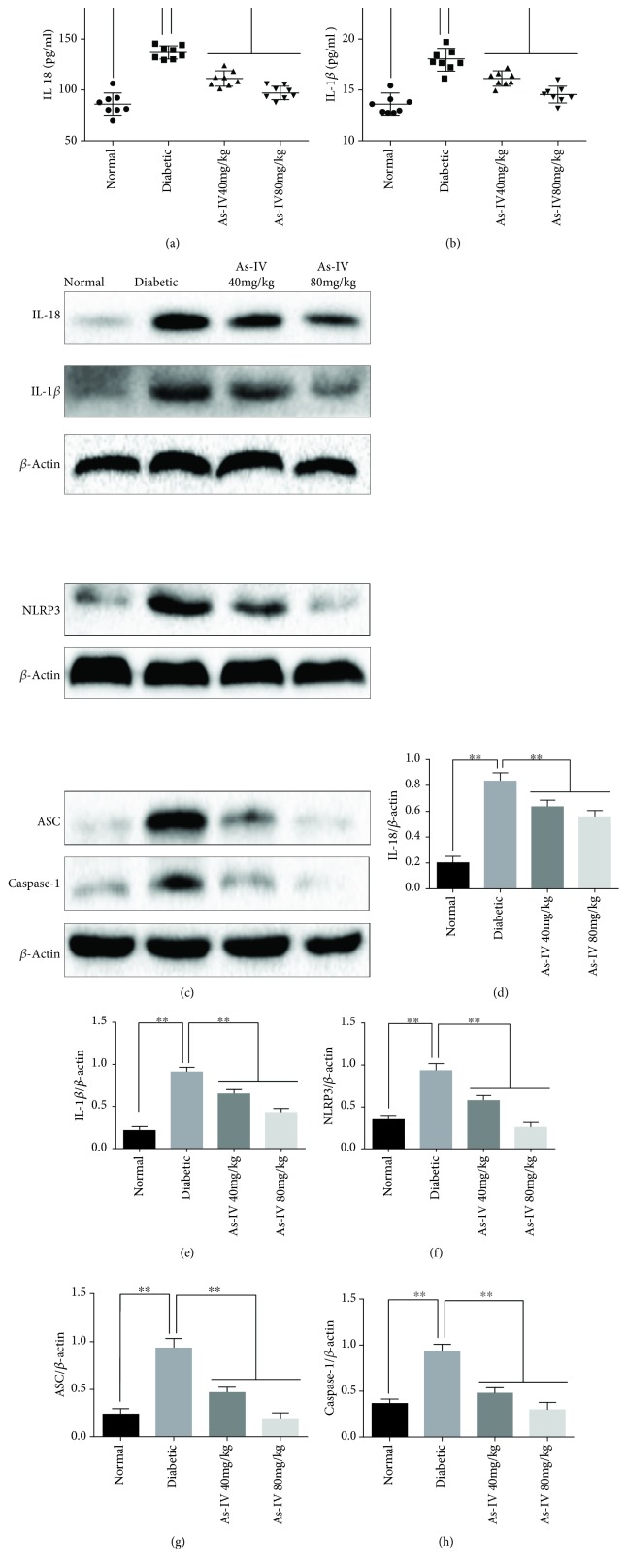
Effects of As-IV on NLRP3, ASC, caspase-1, IL-1*β*, and IL-18 levels in diabetic rats. (a, b) The protein secretion levels of IL-1*β* and IL-18 in the serum of diabetic rats were examined by ELISA. (c-h) NLRP3, ASC, caspase-1, IL-1*β*, and IL-18 expression was detected by western blot in the aorta of diabetic rats. The data are presented as mean ± SD (*n* = 3; ^∗∗^*P* < 0.01).

To determine whether As-IV can inhibit the activation of the NLRP3 inflammasome and subsequent proinflammatory cytokine secretion, protein levels of NLRP3, ASC, caspase-1, IL-1*β*, and IL-18 were assessed by western blot and ELISA. ELISA showed that the secretion of IL-1*β* and IL-18 (Figures 1(a) and 1(b)) dramatically increased in diabetic rats compared with the normal group, and As-IV treatment dramatically reduced IL-1*β* and IL-18 secretions in rat serum. In addition, western blot analysis revealed that the expression of NLRP3, ASC, caspase-1, IL-1*β*, and IL-18 significantly increased in the aorta of diabetic rats and As-IV significantly reduced NLRP3, ASC, caspase-1, IL-1*β*, and IL-18 expression (Figures 1(c)–1(h)).

### 3.2. As-IV Inhibited the Activation of TLR4/NF-*κ*B Pathways in the Aorta of Diabetic Rats

**Figure 2:**
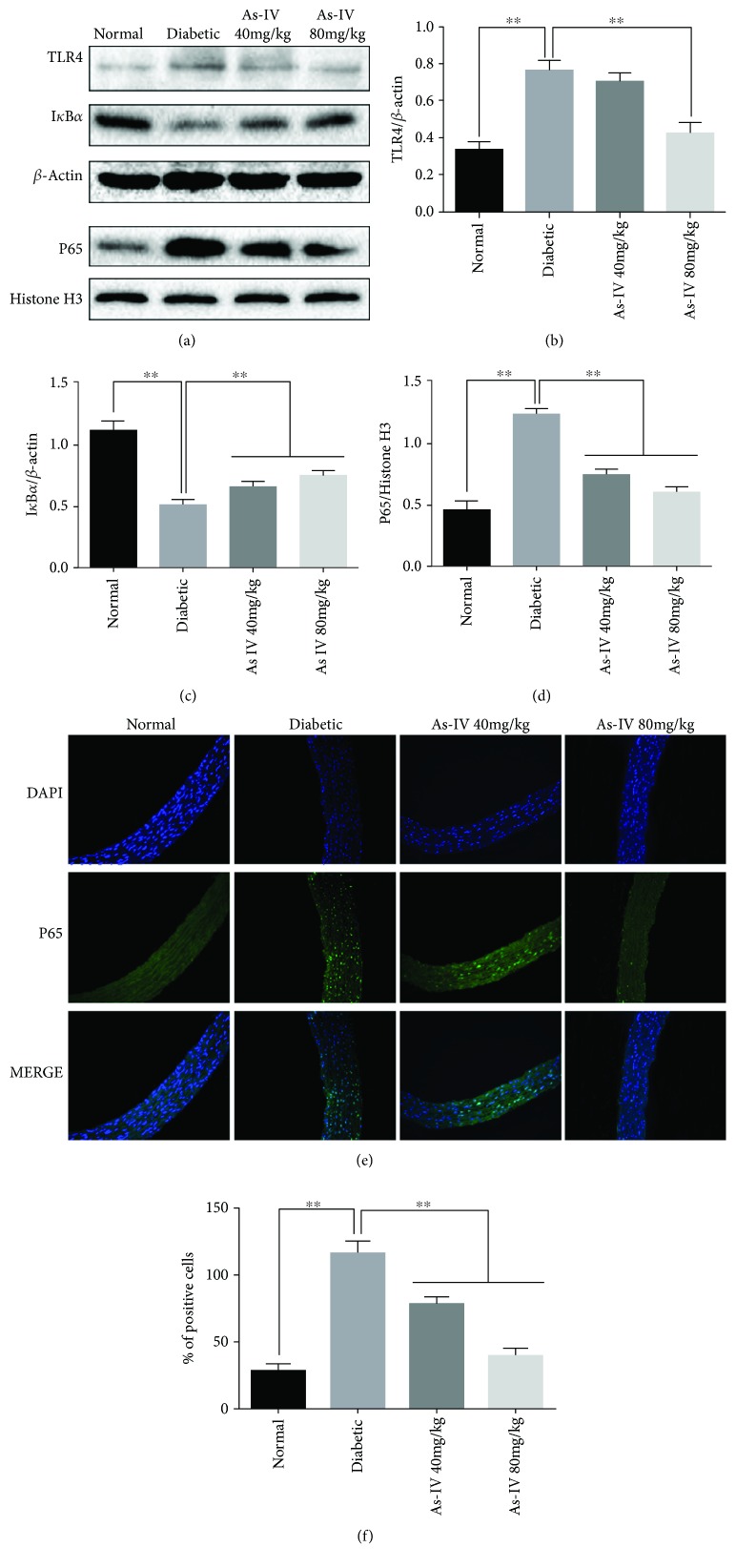
Effects of As-IV on TLR4, I*κ*B*α*, and nucleus NF-*κ*B levels in diabetic rats. (a-d) The levels of TLR4, I*κ*B*α*, and nucleus NF-*κ*B p65 protein were determined by western blot analysis. (e, f) The distribution of NF-*κ*B p65 in the aorta of diabetic rats was examined by immunofluorescence staining. The data are presented as mean ± SD (*n* = 3; ^∗∗^*P* < 0.01).

To determine whether As-IV is involved in the regulation of diabetic-induced activation of the TLR4/NF-*κ*B signaling pathway, western blot and immunofluorescence experiment were performed. The results showed that the protein expression of TLR4 and nucleus NF-*κ*B (Figures 2(a)*–*2(d)) was upregulated and the percentage of NF-*κ*B p65-positive cells was significantly increased (Figures 2(e) and 2(f)), while the expression of I*κ*B*α* was decreased in the diabetic group and all of which were significantly reversed by As-IV.

### 3.3. As-IV Inhibited the Activation of CaSR in the Aorta of Diabetic Rats

**Figure 3:**
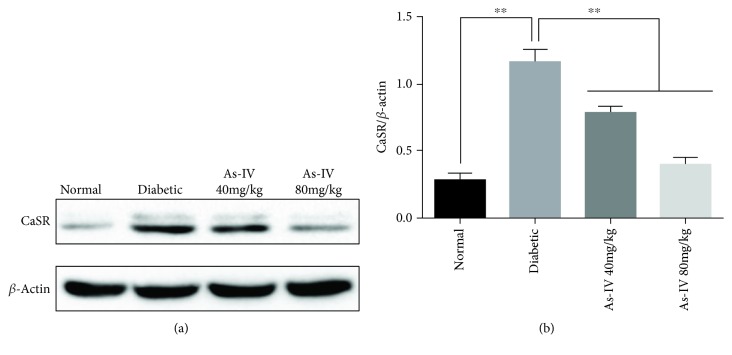
Effects of As-IV on CaSR expression. (a, b) The protein expression of CaSR was detected by western blot. The data are presented as mean ± SD (*n* = 3; ^∗∗^*P* < 0.01).

To evaluate the effect of As-IV on the expression of CaSR in the aorta of diabetic rats, we measured the level of CaSR protein expressions. The results showed that the protein expression of CaSR in diabetic groups was higher than that in the normal group (Figures 3(a) and 3(b)). The elevated level of the CaSR protein expression in the aorta of diabetic rats was reversed by As-IV treatment.

### 3.4. As-IV Inhibited NLRP3 Inflammasome Activation and Subsequent Proinflammatory Cytokine Secretion Induced by High Glucose in HUVECs

**Figure 4:**
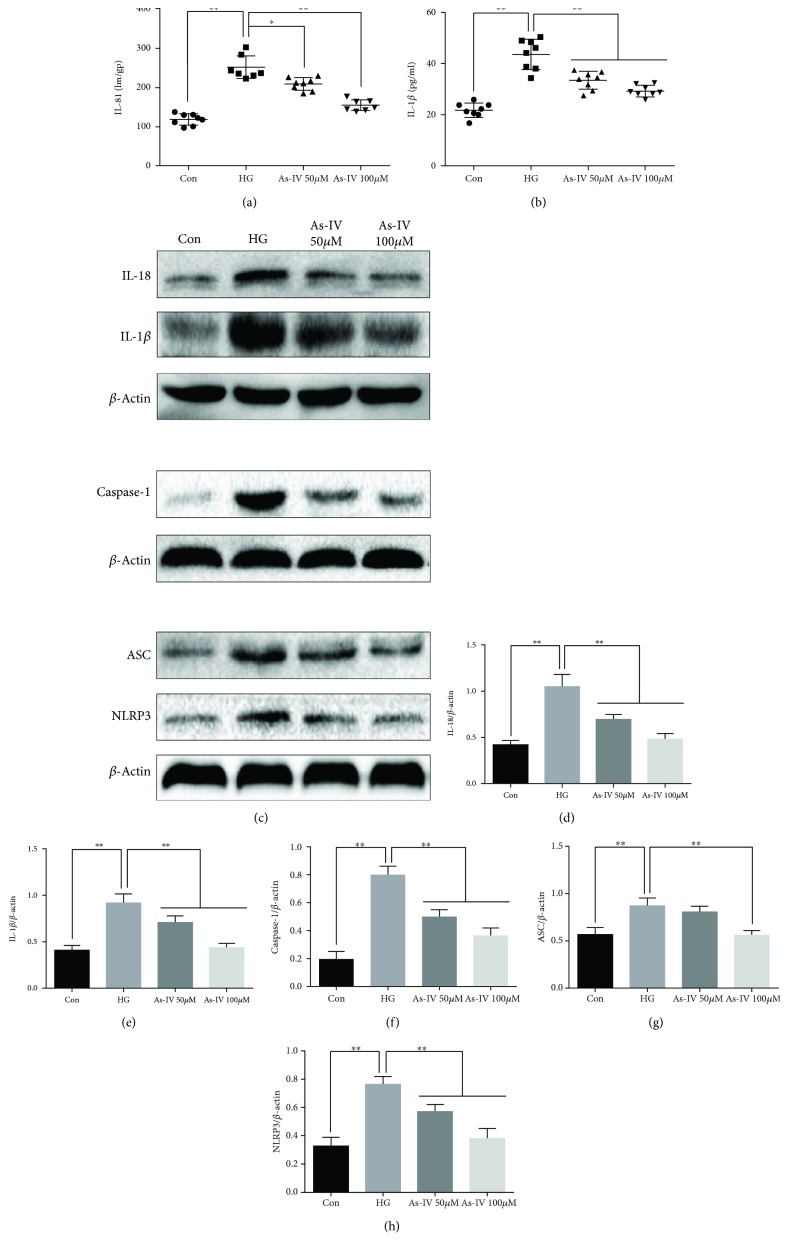
Effects of As-IV on NLRP3, ASC, caspase-1, IL-1*β*, and IL-18 levels in HUVECs. (a, b) IL-18 and IL-1*β* protein levels in HUVEC culture supernatants. (c-h) Western blot was used to detect the protein expression of NLRP3, ASC, caspase-1, IL-1*β*, and IL-18 in HUVECs. The data are presented as mean ± SD (*n* = 3; ^∗^*P* < 0.05; ^∗∗^*P* < 0.01).

To explore whether As-IV inhibits high glucose-induced inflammation via inhibition of NLRP3 inflammasome activation, western blot and ELISA experiment were performed. ELISA showed increased secretion of IL-1*β* and IL-18 in HUVEC culture supernatants after 48 h of high glucose treatment (Figures 4(a) and 4(b)), and As-IV decreased the secretion of IL-1*β* and IL-18. The protein expression levels of NLRP3, ASC, caspase-1, IL-1*β*, and IL-18 were increased by high glucose treatment compared with those of the control (Figures 4(c)*–*4(h)), and administration of As-IV decreased these proteins.

### 3.5. Astragaloside IV Inhibited the Activation of TLR4/NF-*κ*B Pathways Induced by High Glucose in HUVECs

**Figure 5:**
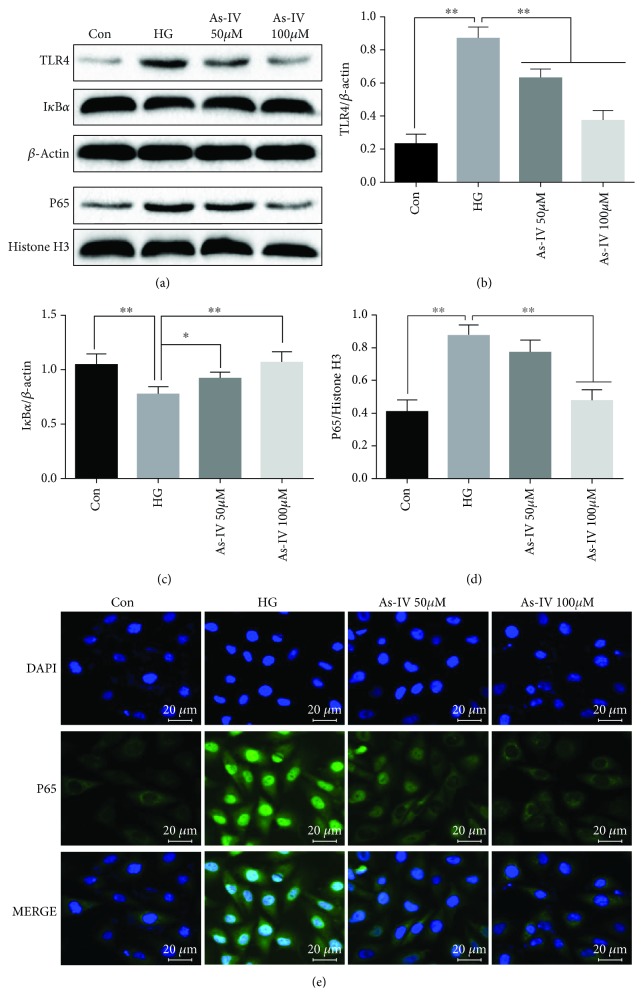
Effects of As-IV on TLR4, I*κ*B*α*, and nucleus NF-*κ*B levels in HUVECs induced by high glucose. (a-d) The protein levels of TLR4, I*κ*B*α*, and nucleus NF-*κ*B were measured by western blot analysis in HUVECs. (e) The translocation of p65 in HUVECs were stained by immunofluorescence. The data are presented as mean ± SD (*n* = 3; ^∗^*P* < 0.05; ^∗∗^*P* < 0.01).

To verify whether As-IV can inhibit the activation of the TLR4/NF-*κ*B signaling pathway induced by high glucose in HUVECs, the protein levels of TLR4 and NF-*κ*B were measured by western blot and immunofluorescence. These results confirmed that administration of As-IV suppressed high glucose-induced increase of TLR4 and NF-*κ*B and decrease of I*κ*B*α* (Figures 5(a)*–*5(d)). Immunofluorescence results showed that NF-*κ*B p65 was mainly expressed in the cytosol of unstimulated HUVECs in the control group (Figure 5(e)); after treatment with high glucose (33 mM), NF-*κ*B p65 was retained in the nucleus and As-IV inhibited the translocation of p65 from the cytosol to the nucleus.

### 3.6. As-IV Inhibited the Activation of CaSR Induced by High Glucose in HUVECs

**Figure 6:**
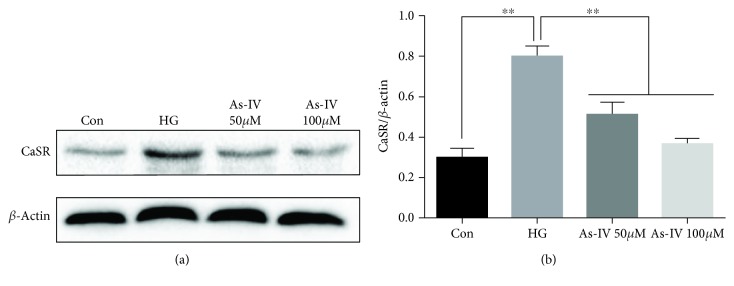
Effects of As-IV on the protein level of CaSR in HUVECs induced by high glucose. (a, b) The protein expression of CaSR was detected by western blot. The data are presented as mean ± SD (*n* = 3; ^∗∗^*P* < 0.01).

As mentioned above, the study found that As-IV can inhibit CaSR activation in high glucose-induced rats. To further confirm whether As-IV can inhibit CaSR activation, western blot experiment was performed. Western blot results showed that high glucose stimulation significantly increased the CaSR level relative to that observed in the control, while treatment with As-IV reduced the protein expression of CaSR in HUVECs (Figures 6(a) and 6(b)).

### 3.7. TLR4/NF-*κ*B and CaSR Play a Critical Role in the Activation of the NLRP3 Inflammasome Induced by High Glucose in HUVECs

**Figure 7:**
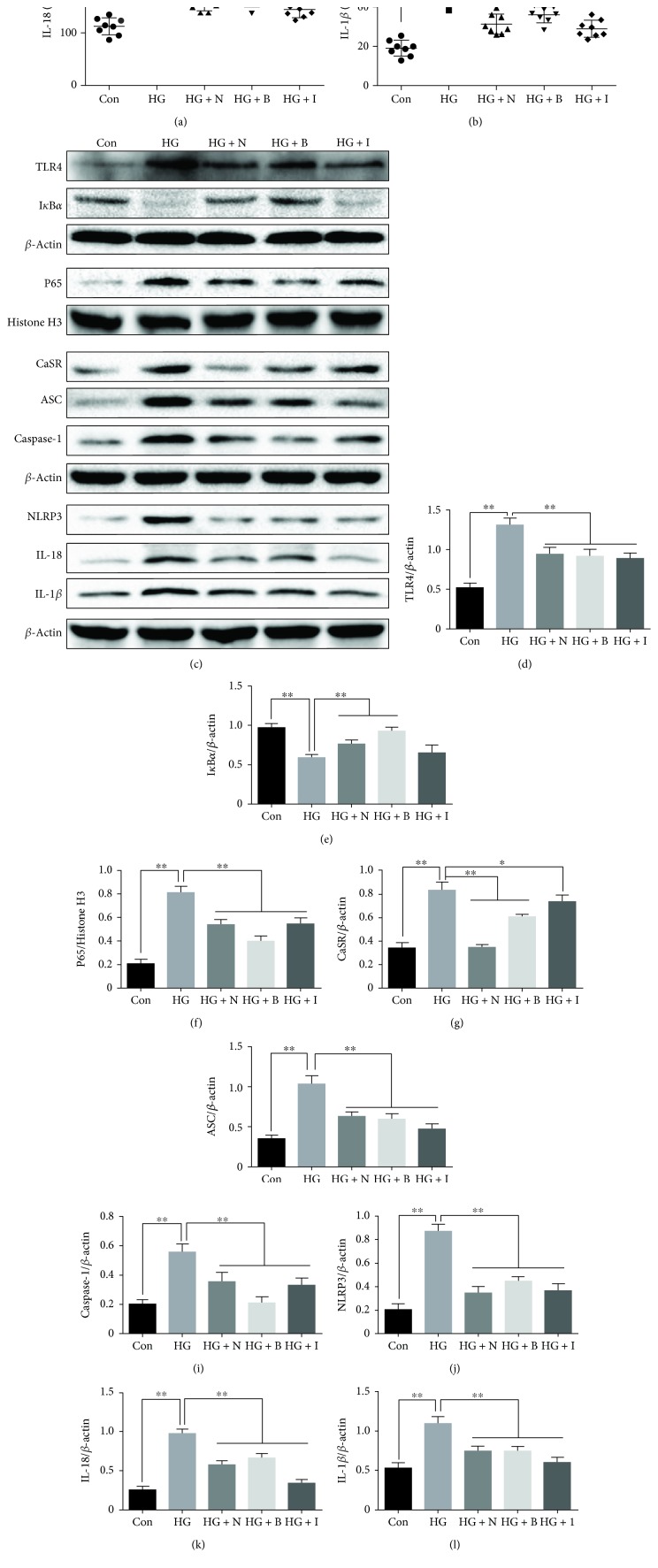
Effects of NPS2143, BAY 11-7082, or INF39 on the expression of NLRP3 inflammasome components in high glucose-stimulated HUVECs. (a, b) The protein secretion levels of IL-1*β* and IL-18 in the cell culture supernatant were examined by ELISA. (c-l) The protein levels of TLR4, I*κ*B*α*, nucleus NF-*κ*B, I*κ*B*α*, CaSR, NLRP3, ASC, caspase-1, IL-1*β*, and IL-18 were measured by western blot analysis in HUVECs. The data are presented as mean ± SD (*n* = 3; ^∗^*P* < 0.05; ^∗∗^*P* < 0.01).

To further determine the role of TLR4/NF-*κ*B and CaSR in the activation of the NLRP3 inflammasome and the interaction between them, HUVECs were cultured with NPS2143, BAY 11-7082, or INF39 for 30 min, and then treated with high glucose (33 mM) for 48 h. The results showed that treatment with NPS2143 (100 nM), BAY 11-7082 (5 *μ*M), or INF39 (10 *μ*M) markedly inhibited high glucose-induced NLRP3, ASC, and caspase-1 (Figures 7(c) and 7(h)*–*7(j)) expression and subsequently decreased the expression of IL-1*β* and IL-18 in HUVECs (Figures 7(a) and 7(b)). Meanwhile, we also found that NPS2143 also downregulated the expression of TLR4 and NF-*κ*B (Figures 7(c)*–*7(f)) and BAY 11-7082 decreased the expression of CaSR (Figures 7(c) and 7(g)) in HUVECs. In addition, INF39 also inhibited the expression of TLR4, NF-*κ*B, and CaSR (Figures 7(c)*–*7(g)).

### 3.8. CaSR Participates in the Activation of the NLRP3 Inflammasome in HUVECs

**Figure 8:**
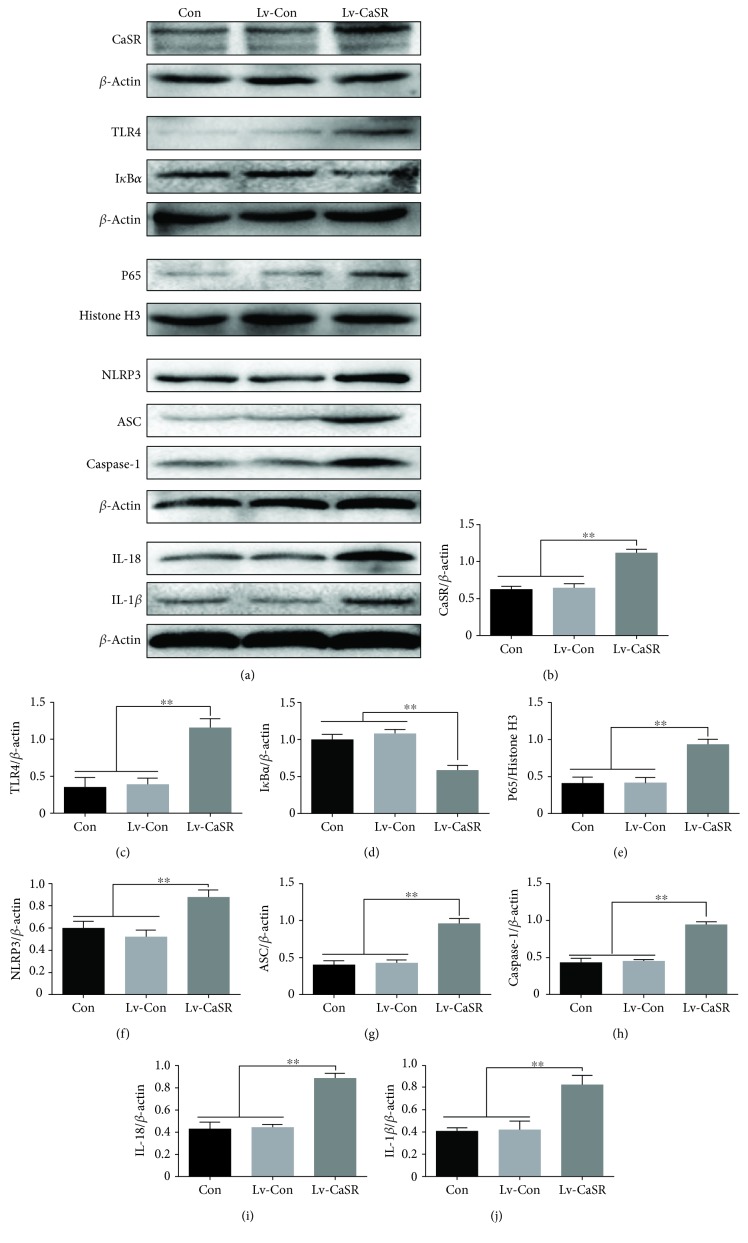
Effects of CaSR overexpression on CaSR, TLR4, nucleus NF-*κ*B, I*κ*B*α*, NLRP3, ASC, caspase-1, IL-1*β*, and IL-18 levels in HUVECs. (a-j) HUVECs infected with LV-Con or LV-CaSR, and the protein expression levels of CaSR, TLR4, nucleus NF-*κ*B, I*κ*B*α*, NLRP3, ASC, caspase-1, IL-1*β*, and IL-18 levels were determined by western blot. The data are presented as mean ± SD (*n* = 3; ^∗∗^*P* < 0.01).

In order to further explore whether CaSR overexpression is involved in the activation of the NLRP3 inflammasome, HUVECs were infected with LV-Con or LV-CaSR, and western blotting was performed. After stable transfection, the expression of CaSR in the LV-CaSR group was significantly higher than those in the control group and the LV-Con group (Figures 8(a) and 8(b)). Furthermore, the overexpression of CaSR markedly increased TLR4, NF-*κ*B p65, NLRP3, ASC, caspase-1, IL-1*β*, and IL-18 expression (Figures 8(a) and 8(c)*–*8(j)).

### 3.9. TLR4 Is Involved in High Glucose-Induced NLRP3 Inflammasome Activation in HUVECs

**Figure 9:**
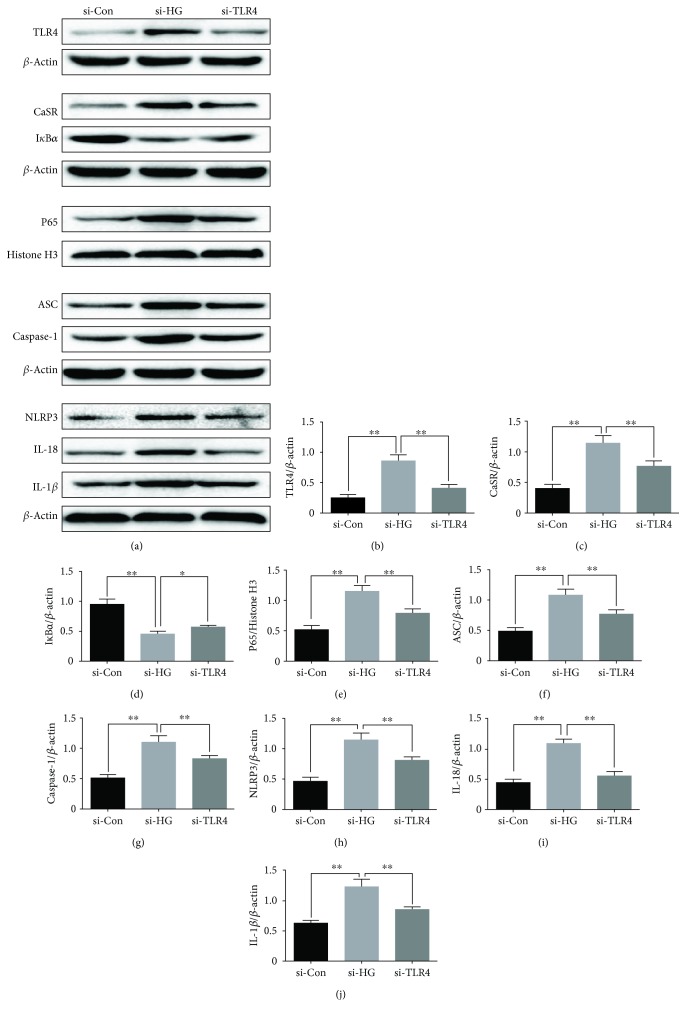
Effect of high glucose and TLR4 siRNA on CaSR, TLR4, nucleus NF-*κ*B, I*κ*B*α*, NLRP3, ASC, caspase-1, IL-1*β*, and IL-18 levels in HUVECs. (a-j) HUVECs were transfected with scrambled or TLR4 siRNA and incubated with high glucose, and the protein expression levels of CaSR, TLR4, nucleus NF-*κ*B, I*κ*B*α*, NLRP3, ASC, caspase-1, IL-1*β*, and IL-18 levels were determined by western blot. The data are presented as mean ± SD (*n* = 3; ^∗^*P* < 0.05; ^∗∗^*P* < 0.01).

To better understand the effect of TLR4 siRNA in high glucose-induced NLRP3 inflammasome activation, the cells were transfected with TLR4 or scramble siRNA. We found that transfection of TLR4 siRNA into HUVECs downregulated the expression of CaSR, nucleus NF-*κ*B, NLRP3, ASC, caspase-1, IL-1*β*, and IL-18 levels in a high glucose environment (Figures 9(a)*–*9(j)).

## 4. Discussion

Vascular endothelial cells (VECs) are present in the entire circulatory system, from the smallest capillaries to the heart. Although originally thought to be a simple mechanical barrier between the vascular wall and blood, VECs play an important role in maintaining vascular structure and function [3]. Hyperglycemia-induced inflammation is thought to be a major risk factor of endothelial cell dysfunction and increased risk of cardiovascular events [29].

Inflammation is an important immune response against an infection or an injury and is involved in the occurrence and development of various diseases [30, 31], including cardiovascular diseases, such as atherosclerosis [32], atrial fibrillation [33], hypertension [34], and arterial aging [35]. As a risk factor for cardiovascular disease, there is a close relationship between inflammation and cardiovascular events [36]. Meanwhile, inflammation plays a critical role in diabetic vascular complications [37]. IL-1*β* and IL-18 are a product of NLRP3 inflammasome activation, both produced by macrophages and monocytes, but also derived from other cell types, such as VECs [38]. IL-18 belongs to the IL-1 superfamily, which plays an important role in inflammatory cascade and atherosclerosis [39]. As a proinflammatory cytokine, IL-18 has atherogenic properties and is highly expressed in atherosclerotic plaques [40]. IL-1*β* is a major player in autoinflammatory diseases and is also a key promoter of tissue and systemic inflammation in diabetes mellitus [41]. Since IL-1*β* and IL-18 are both promoters of immune response, as well as direct proinflammatory cytokines, reducing IL-1*β* and IL-18 activity in some diseases is a good strategy. In our study, we examined the expression of and the content of IL-1*β* and IL-18 in vivo and in vitro. Our research shows that treatment with As-IV inhibits the release and expression of IL-18 and IL-1*β*. NLRP3 inflammasome activation contributes to the development of diabetic vascular endothelial dysfunction [14]. In addition, the secretion of biologically active IL-18 and IL-1*β* requires the activation of the NLRP3 inflammasome to exert their biological effects. Therefore, we investigated the effect of As-IV on the activation of the NLRP3 inflammasome. Our study found that As-IV inhibits the expression of NLRP3, ASC, and caspase-1 in vivo and in vitro. Furthermore, in order to investigate whether the NLRP3 inflammasome is involved in the regulation of IL-1*β* and IL-18, we used the NLRP3 irreversible inhibitor INF39 to examine whether inflammasome blockade alters the expression of IL-1*β* and IL-18. Our data indicated that inhibition of the NLRP3 inflammasome reduces the expression of IL-1*β* and IL-18.

As a classical signaling pathway of inflammation, the TLR4/NF-*κ*B signaling pathway not only participates in the regulation of inflammation [42] but also participates in the activation of the NLRP3 inflammasome [6]. Toll-like receptors (TLRs) are membrane proteins and are associated with the occurrence of cardiovascular dysfunctions such as atherosclerosis, ischemic heart disease, heart failure, and cerebrovascular injury [43, 44]. TLR4 belongs to the toll-like receptor family, and it is the most investigated receptor in this area. After activation of TLR4, NF-*κ*B p65, which is in the resting state in the cytoplasm, is separated from its inhibitory protein I*κ*B*α* and transferred into the nucleus to promote the secretion of proinflammatory cytokines [45, 46]. In the current study, our results indicate that As-IV treatment decreased the expressions of TLR4 and nucleus NF-*κ*B p65 and increased the I*κ*B*α* expression. Moreover, As-IV can also promote the translocation of NF-*κ*B p65 from the nucleus to the cytoplasm which is consistent with previous research [47]. CaSR not only participates in the regulation of inflammation but also plays an important role in the activation of the NLRP3 inflammasome [19, 48]. Meanwhile, CaSR may also participate in the development of diabetic macroangiopathy by promoting apoptosis in high glucose-treated cells [49]. Therefore, we investigated the effect of As-IV on CaSR. The result shows that As-IV can reverse the increased CaSR induced by high glucose.

In addition, in this experiment, we not only explored the effects of As-IV on the activation of TLR4, NF-*κ*B, CaSR, and NLRP3 inflammasome but also discussed the role of TLR4, NF-*κ*B, and CaSR in the activation of the NLRP3 inflammasome. Huang et al.'s experimental results showed that TLR4 participates in the regulation of the NLRP3 inflammasome in H9C2 cells [50]. In this study, high glucose-induced NLRP3 inflammasome activation and increased expression of CaSR in HUVECs was abolished by TLR4 silencing. NF-*κ*B pathway blocker BAY 11-7082 has previously been used to block the NLRP3 inflammasome [50, 51] and reverse the increased expressions of CaSR [52]. It is worth noting that the regulation of the NLRP3 inflammasome and NF-*κ*B is governed by CaSR [52, 53]. In this experiment, we first reported the role of CaSR in NLRP3 activation in endothelial cells. We found that BAY 11-7082 not only inhibits the expression of CaSR and TLR4 but also inhibits the activation of the NLRP3 inflammasome. Similarly, NPS2143 can inhibit the activation of TLR4/NF-*κ*B and the NLRP3 inflammasome. Meanwhile, in this study, we first discussed the effect of INF39 on CaSR and NF-*κ*B. The results showed that INF39, a key component inhibitor of the NLRP3 inflammasome, can block not only NLRP3 activation but also the TLR4/NF-*κ*B pathway and CaSR. It is worth emphasizing that the effect of CaSR on the NLRP3 inflammasome has been proven again in stable transfection experiments.

## 5. Conclusions

**Figure 10:**
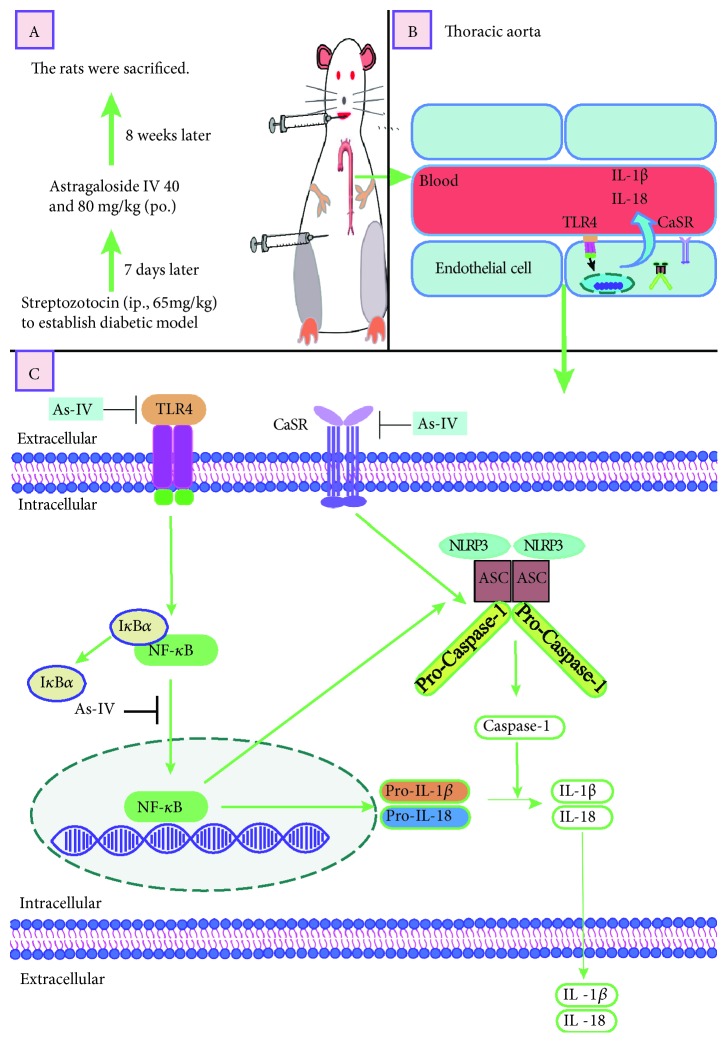
The proposed mechanism of As-IV for the inhibition of NLRP3 inflammasome activation.

In summary, our work showed that As-IV could inhibit high glucose-induced production of IL-1*β* and IL-18 and the underlying molecular mechanisms of As-IV may be inhibiting NLRP3 inflammasome activation via inhibition of the TLR4/NF-*κ*B pathway and CaSR (Figure 10).

## Data Availability

The data used to support the findings of this study are available from the corresponding author upon request.
